# LncRNA KCNQ1OT1 sponges miR-15a to promote immune evasion and malignant progression of prostate cancer via up-regulating PD-L1

**DOI:** 10.1186/s12935-020-01481-8

**Published:** 2020-08-15

**Authors:** Qi-Hua Chen, Bo Li, De-Guo Liu, Biao Zhang, Xian Yang, Ya-Ling Tu

**Affiliations:** 1 Department of Andrology, The First Hospital, Hunan University of Chinese Medicine, No.95, Shaoshan Middle Road, Yuhua District, Changsha, 410007 Hunan People’s Republic of China; 2 Graduate School, Hunan University of Chinese Medicine, Changsha, 410208 People’s Republic of China; 3 Department of Dermatology, The First Hospital, Hunan University of Chinese Medicine, Changsha, 410007 People’s Republic of China

**Keywords:** Prostate cancer, KCNQ1OT1, MiR-15a, PD-L1, Immune evasion

## Abstract

**Background:**

We focused on the KCNQ1OT1/miR-15a/PD-L1 axis and explored its significance in regulating immune evasion and malignant behaviors of prostate cancer (PC) cells.

**Methods:**

The expression levels of KCNQ1OT1, miR-15a, PD-L1, and CD8 in cells or tissues were examined by RT-qPCR, western blot or immunohistochemistry (IHC) assays. The direct regulations between KCNQ1OT1, miR-15a and PD-L1 were validated by luciferase reporter assay. PC cells were co-cultured with CD8^+^ T cells to study the immune evasion. Proliferation, apoptosis, migration and invasion abilities were detected by MTT, flow cytometry, wound healing and Transwell assays, respectively. The cytotoxicity of CD8^+^ T cells was determined by LDH cytotoxicity Kit. Epithelial–mesenchymal transition (EMT) and Ras/ERK signaling markers were evaluated by western blot.

**Results:**

KCNQ1OT1, PD-L1 and CD8 were increased, while miR-15a was decreased in PC tissues. MiR-15a directly bound to the 3′-UTR of PD-L1 and inhibited the expression of PD-L1. Overexpressing miR-15a in PC cells was sufficient to promote cytotoxicity and proliferation, while inhibit apoptosis of CD8^+^ T cells, and also suppressed viability, migration, invasion and EMT while promoted apoptosis of PC cells. The above anti-tumor effects of miR-15a were reversed by overexpressing PD-L1. KCNQ1OT1 sponged miR-15a and released its inhibition on PD-L1. Functionally, KCNQ1OT1 in PC cells was essential for suppressing the cytotoxicity of CD8^+^ T cells and maintaining multiple malignant phenotypes of PC cells. The Ras/ERK signaling was suppressed after overexpressing miR-15a or knocking down KCNQ1OT1.

**Conclusions:**

LncRNA KCNQ1OT1 sponges miR-15a to promote immune evasion and malignant progression of PC via up-regulating PD-L1.

## Background

Prostate cancer (PC), with its high morbidity as well as mortality, is a global threat to male health [1, 2]. Although ranking after some other cancers, such as lung cancer, liver cancer, and gastric cancer, both the incidence and the mortality of PC remain on the rise in China [3], potentially due to the delayed presentation of most symptoms and the limited accessibility of PC screening. Despite the fact that androgen deprivation proves effective for patients with advanced PC, the effect is only temporary in most cases and fails to prevent the relentless progression into the lethal metastatic castration-resistant PC (mCRPC) [4]. Cell autonomous behaviors, such as proliferation, migration, invasion and epithelial–mesenchymal transition (EMT), critically drive the malignant growth, the metastatic spread, and the resistance to therapy of PC. In addition, these biological behaviors also link tumor cells with other cell types within the tumor microenvironment to nurture tumor development [5]. One mechanism utilized by tumor cells is through the regulation of programmed death ligand 1 (PD-L1). On one hand, the high level of PD-L1 promotes tumor proliferation, invasion, angiogenesis, cancer stemness and EMT [6–8]. On the other hand, PD-L1 from tumor cells engages its cognate receptor PD-1 on CD8^+^ cytotoxic T cells, initiating negative signaling and tumor evasion [9]. As a result, targeting PD-L1/PD-1 axis has become an intriguing immunotherapeutic strategy and has proved its clinical value in a variety of human cancers, such as melanoma [10, 11], non-small-cell lung cancer [12], hepatocellular carcinoma [13], gastric cancer [14], and etc.

Unfortunately, mCRPC patients presented de novo resistance to PD-L1/PD-1 axis blockade [15, 16], driving research to understand the underlying mechanisms and to develop therapies to overcome this resistance. Martin et al. showed that although PC cell lines could up-regulate PD-L1 in response to inflammatory cytokines, its expression was rare in primary prostate tumors [17]. Yet another study reported that PD-L1 expression was significantly associated with CD8^+^ T-cell density, and both were biomarkers for high risk of disease progression in node-positive PC patients [18]. At this stage, it is not clear what controls PD-L1/PD-1 axis and subsequent cytotoxic activities of CD8^+^ T cells in PC, the understanding of which will help to develop novel strategies that may boost the effect of PD-L1/PD-1 axis blockade. Our bioinformatic analysis using the Starbase platform (http://starbase.sysu.edu.cn/index.php) revealed potential binding sites to miR-15a within the 3′-untranslated region (3′-UTR) of human PD-L1 gene, suggesting that miR-15a might directly target PD-L1. MiR-15a is a well-characterized tumor suppressor that targets multiple oncogenes such as BCL2, MCL1, CCND1, and WNT3A [19]. MiR-15a shares the same seed sequence with miR-16 and both are derived from a gene cluster on chromosome 13q14 [20]. MiR-15a/miR-16 gene cluster is a well-characterized tumor suppressor that targets a variety of cancer-related phenotypes, including proliferation, apoptosis, invasion, angiogenesis and chemoresistance [20]. In PC, the miR-15a/miR-16 gene cluster not only targets multiple oncogenic activities, but also controls tumor microenvironment crosstalk [21, 22]. No study, however, has established the link between miR-15a and PD-L1 or the functional significance of their interaction in cancers.

Besides showing that miR-15a may bind to the 3′-UTR of PD-L1, our bioinformatic analysis using Starbase platform also revealed the potential binding of a long non-coding RNA (lncRNA) KCNQ1OT1 to the same sequence of miR-15a, implying that KCNQ1OT1 may sponge miR-15a, which then releases the suppression of miR-15a on PD-L1. Cumulative evidence suggests that KCNQ1OT1 promotes oncogenic phenotypes and chemoresistance of multiple cancers, including colon cancer, lung cancer, breast cancer, hepatocellular carcinoma, and tongue cancer [23–27]. However, few studies have explored the functional significance of KCNQ1OT1 in PC or the regulation between KCNQ1OT1 and miR-15a so far.

To test whether the KCNQ1OT1/miR-15a/PD-L1 axis may function and contribute to the malignant phenotypes as well as immune evasion of PC, we compared the expression levels of these three molecules between PC and normal prostate tissues and analyzed the correlation among them. Next, we used two different PC cell lines, DU145 and PC-3, and examined the functional significance of the KCNQ1OT1/miR-15a/PD-L1 axis on malignant behaviors as well as the cytotoxicity of CD8^+^ T cells. Our study reveals that KCNQ1OT1 sponges miR-15a and subsequently up-regulates PD-L1, which not only thwarts the cytotoxicity of CD8^+^ T cells but also promotes the malignant progression of PC. This is the first study supporting the significance of the KCNQ1OT1/miR-15a/PD-L1 axis in immune evasion and PC progression.

## Materials and methods

### Human tissues

Thirty pairs of PC tissues and matching normal prostate tissues were obtained from PC patients during surgery and frozen immediately in liquid nitrogen till further use. Protocols involving human sample collection were reviewed and approved by the Ethics Committee of the First Hospital of Hunan University of Chinese Medicine (Changsha, Hunan, China). A written consent was signed by each participant.

### Reverse transcription quantitative real-time PCR (RT-qPCR)

Trizol reagent (Invitrogen, Carlsbad, CA, USA) was used to extract total RNA from either cells or tissues. Total RNA was then reversely transcribed into cDNA using Takara reverse transcription system (Dalian, China). qPCR analysis was performed with Applied Biosystems 7500 Real Time PCR System (Thermo Fisher Scientific, Waltham, MA, USA) using iQ™ SYBR^®^ Green Supermix Kit (Bio-Rad, Hercules, CA, USA). GAPDH and U6 were detected as the internal controls for mRNA and miRNA, respectively. Primers for KCNQ1OT1, miR-15a, PD-L1 and CD8 were purchased from Sangon Biotech (Shanghai, China). Target gene expressions were calculated using the 2^−^^ ΔΔCt^ method [28].

### Immunohistochemistry (IHC)

Paraffin sections of 4 µm in thickness were prepared from human tissues fixed in 10% neutral formalin. For detection of CD8, tissue sections were de-paraffinized in xylene, rehydrated in diluted alcohol series, blocked with 0.3% H_2_O_2_ followed by 5% normal goat serum, and incubated with anti-CD8 primary antibodies (1:200, ab4055, Abcam, Cambridge, MA, USA) at 4 °C overnight. After incubating the tissues with biotinylated anti-rabbit secondary antibody (BP-9100, Vector Labs, Burlingame, CA, USA) at room temperature for 30 min, the signal was amplified using Vectastain ABC-HRP solution (Vector Labs) and developed using Diaminobenzidine (DAB) substrate (Vector Labs). At the end, the slides were counterstained with hematoxylin.

### Cell culture

The human PC cell lines DU145 and PC-3 were purchased from the American Type Culture Collection (ATCC, Manassas, VA, USA) and cultured in Dulbecco’s modified Eagle’s medium (DMEM, Gibco, Gaithersburg, MD, USA) supplemented with 10% fetal bovine serum (FBS, Hyclone, Logan, UT, USA) and 1% penicillin and streptomycin in a humidified atmosphere containing 5% CO_2_ at 37 °C.

### Cell transfection

The hsa-miR-15a mimics, hsa-miR-15a inhibitor, the corresponding controls (mimics NC and inhibitor NC), shRNA specifically targeting KCNQ1OT1 (shKCNQ1OT1), and the corresponding control shRNA (shNC) were designed and purchased from GenePharma (Shanghai, China). KCNQ1OT1 or PD-L1 cDNA was cloned into pcDNA3.1 vector (ThermoFisher, Waltman, MA, USA) to achieve their overexpression, and the empty vector was used as the negative control (pcDNA3.1-NC). The transfection of above oligonucleotides (miR-15a mimics/inhibitor/mimics NC/inhibitor NC: 100 nM) or vectors (shKCNQ1OT1/pcDNA3.1-KCNQ1OT1/pcDNA3.1-PD-L1/shNC/pcDNA3.1-NC: 50 nM) was performed using Lipofectamine 2000 (Invitrogen, Carlsbad, CA, USA) according to the manufacturer’s instructions.

### Western blot

Total protein was extracted from cells using RIPA buffer and protein concentration was measured using the BCA Protein Assay Kit (Sangon Biotech). After separated by SDS-PAGE, proteins were transferred onto PVDF membranes (Millipore, Billerica, MA, USA), then blocked with 5% nonfat milk in TBST at room temperature for 1 h. The membranes were then incubated with the following primary antibodies (all from Cell Signaling Technology, Danvers, MA, USA): PD-L1 (1:1000, #13,684), Ras (1:1000, #3965), p-ERK1/2 (1:1000, #8544), ERK1/2 (1:1000, #4695), Snail (1:1000, #3879), E-cadherin (1:1000, #3195), N-cadherin (1:1000, #13,116), MMP-9 (1:1000, #13,667), or GAPDH (internal control; 1:3000, #5174) at 4 °C overnight. Upon incubating the membrane with HRP-conjugated anti-rabbit secondary antibody (1:5000, #5127) at room temperature for 2 h, the signal was developed using the ECL system (Beyotime, Jiangsu, China). Relative protein levels were determined by band intensities quantitated using the Image J software.

### Luciferase reporter assay

Starbase website (http://starbase.sysu.edu.cn/index.php) was used to identify the potential binding sites of miR-15a on PD-L1 and KCNQ1OT1. The wild type (WT) or mutated (MUT) binding sequences of human PD-L1 and KCNQ1OT1 gene were cloned into pRL-CMV luciferase reporter plasmid and co-transfected into DU145 or PC-3 cells with miR-15a mimics, miR-15a inhibitor, or the corresponding controls using Lipofectamine 2000 (Invitrogen). After 48 h, the luciferase activity was detected using the Dual Luciferase Reporter Assay System (Promega, Madison, WI, USA) following the manufacturer’s instructions.

### Isolation of human CD8^+^ effector T cells

CD8^+^ T cells were isolated from whole blood of healthy individuals using EasySep™ Direct Human CD8^+^ T cell Isolation Kit (STEMCELL, Cambridge, MA, USA) according to the manufacturer’s instructions. Isolated CD8^+^ T cells were cultured with RPMI-1640 medium (Gibco, Gaithersburg, MD, USA) containing 10% FBS before used for further experiments.

### Lactate dehydrogenase (LDH) cytotoxicity assay

The cytotoxicity of CD8^+^ T cells was examined using CyQUANT™ LDH Cytotoxicity Assay Kit (Thermo Fisher Scientific) according to the manufacturer’s protocol. Briefly, isolated effector CD8^+^ T cells were co-cultured with target PC cells at different ratios at 37 °C for 24 h. The conditioned medium from each experimental condition was transferred into a 96-well plate in triplicate and incubated with the reaction mixture at room temperature for 30 min. Upon adding the stop solution, OD490 and OD680 values were measured, and the difference (OD490–OD680) represented the LDH activity. The cytotoxicity of CD8^+^ T cells was calculated as: % Cytotoxicity = [(Compound-treated LDH activity-Spontaneous LDH activity)/(Maximum LDH activity-Spontaneous LDH activity)] × 100%.

### Proliferation of CD8^+^ T cells labeled with carboxyfluorescein succinimidyl ester (CFSE)

The proliferation of CD8^+^ T cells upon co-culture with PC cells was detected using CFSE labelling as described previously [29]. Briefly, effector CD8^+^ T cells were stimulated with anti-CD3 and anti-CD28 monoclonal antibodies (BD Biosciences, San Jose, CA, USA). Then cells were labeled with CFSE (Invitrogen), and co-cultured with target PC cells at 37 ºC for 24 h. Cells after indicated treatment were harvested and the proliferation of CD8^+^ T cells was examined by flow cytometry on FACSCalibur (BD Biosciences, San Jose, CA, USA).

### Apoptosis analysis with Annexin V/PI staining

Apoptotic cells were detected using Annexin V-FITC Apoptosis Detection Kit (Thermo Fisher Scientific) as instructed by the manufacturer. Briefly, cells were seeded in 24-well plates and cultured for approximately 24 h to reach a confluence of nearly 80%. Cells were then stained with the Annexin V-FITC and PI solution for 15 min, and analyzed using the FACSCalibur to determine the percentage of apoptotic cells, which were represented as the percentage sum of early apoptotic (Annexin V^+^PI^−^) and late apoptotic (Annexin V^+^PI^+^) cells.

### 3-(4, 5-dimethylthiazolyl-2)-2, 5-diphenyltetrazolium bromide (MTT) assay

The cell viability was determined using MTT assay. Briefly, target cells (2 × 10^4^ cells/mL) were seeded in 96-well plates and cultured for 24, 48, or 72 h, respectively. Then 20 µL of MTT solution (5 mg/mL in PBS; Sigma) was added to each well for a further 4 h at 37 ºC. Upon removing the medium, DMSO (100 µL/well) was added to dissolve formazan crystals formed in live cells and the optical density was assessed with a Bio-Rad 550 microplate reader (Bio-Rad, Hercules, CA, USA) at 490 nm.

### Wound healing migration assay

The wound healing assay was performed as described previously [30]. Briefly, PC cells were plated into 24-well plate, grew to 90% confluence, and washed with serum-free medium. A scratch was then made across the cell layer using a sterile pipette tip and the wound was photographed immediately (0 h) and 24 h later at the same location, with the width (W) of the scratch measured. The migration rate was calculated as (W_0h _− W_24h_)/W_0h_ **×** 100%.

### Transwell invasion assay

Cell invasion was assessed using Corning Biocoat™ Invasion Chamber (Corning, Lowell, MA, USA) following the manufacturer’s protocol. PC cells suspended in serum-free DMEM were seeded into the top well of the chamber (1 × 10^5^ cells/well) and DMEM containing 10% FBS (500 µL/well) was added into the lower chamber. After 24 h, the invaded cells on the lower side of the membrane were fixed in 95% methanol, stained with crystal violet, and photographed under an inverted microscope.

### Statistical analysis

Statistical analysis was performed using SPSS 13.0 software. All quantitative data were presented as mean ± standard deviation (SD) from three independent experiments. Comparison between two groups was performed using the Student’s *t* test and that among three or more groups using one-way analysis of variance (ANOVA) followed by Tukey’s post hoc test. The correlations between KCNQ1OT1, miR-15a, PD-L1 and CD8 in PC tissues were analyzed using Spearman correlation analysis. A *P* value of < 0.05 was considered statistically significant.

## Results

### KCNQ1OT1, PD-L1 and CD8 were up-regulated, while miR-15a down-regulated in PC tissues

**Fig. 1:**
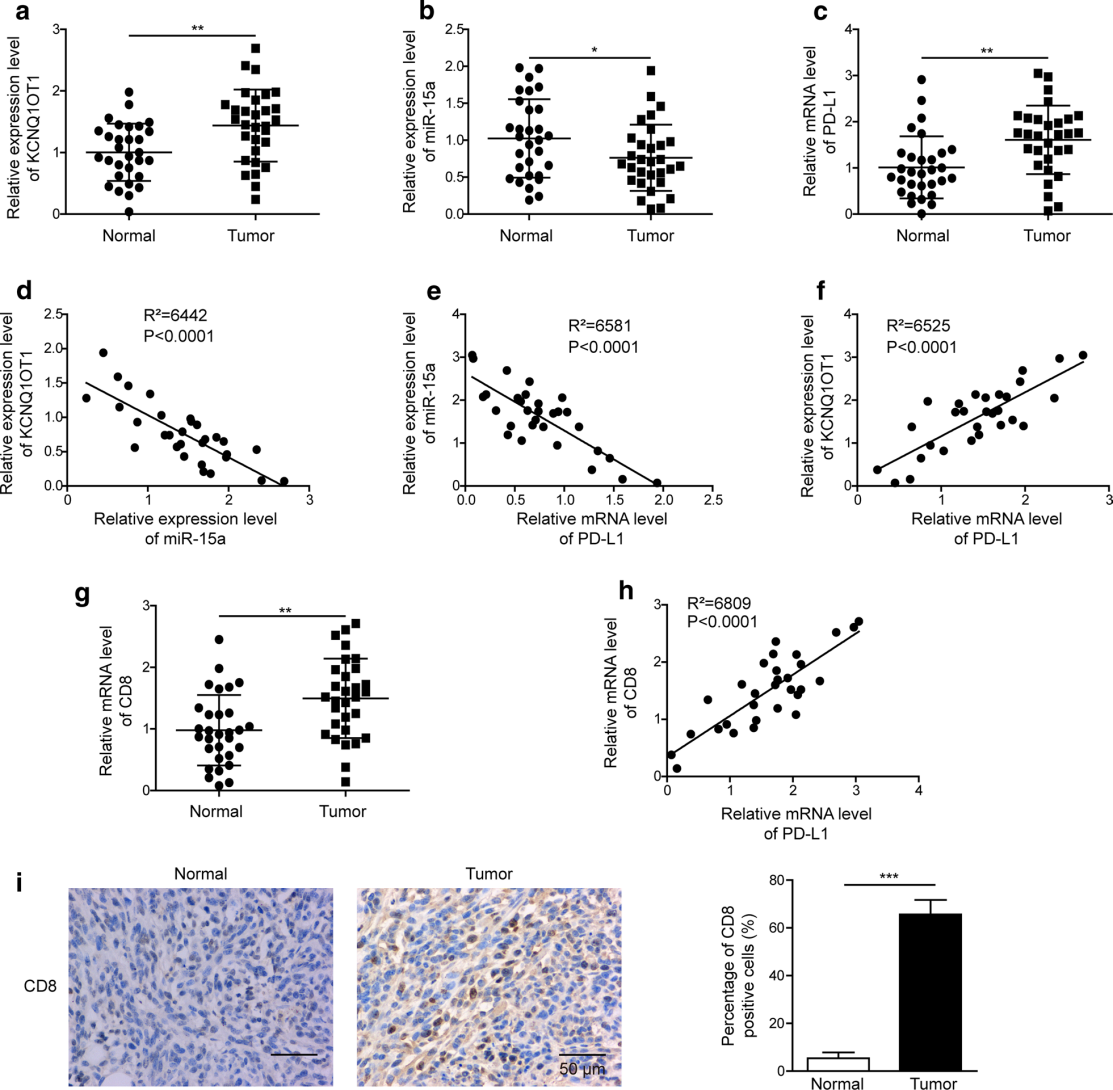
KCNQ1OT1, PD-L1 and CD8 were up-regulated, while miR-15a was down-regulated in PC tissues. **a**–**c** The expression levels of KCNQ1OT1 (**a**), miR-15a (**b**) and PD-L1 (**c**) were examined by RT-qPCR and compared between 30 pairs of PC tissues and the matching para-tumor normal tissues. **d**–**f** The correlations between KCNQ1OT1 and miR-15a (**d**), between miR-15a and PD-L1 (**e**), and between KCNQ1OT1 and PD-L1 (**f**) in PC tissues were examined using Spearman correlation analysis. **g** CD8 mRNA levels were examined by RT-qPCR and compared between 30 pairs of PC tissues and the matching para-tumor normal tissues. **h** The correlation between CD8 and PD-L1 in PC tissues was examined using Spearman correlation analysis.** i** CD8 protein level was examined by IHC. Representative images from PC tissue and the matching normal tissues were shown on the left and quantification of CD8 positive cells on the right. Scale bar: 50 µm. *P < 0.05, **P < 0.01 and ***P < 0.001

To examine the potential crosstalk among KCNQ1OT1, miR-15a, PD-L1, and CD8^+^ cytotoxic T cells in PC, we compared the expression levels of these molecules between 30 pairs of PC tissues and matching para-tumor normal tissues. By RT-qPCR analysis, we detected significantly higher KCNQ1OT1 (Fig. 1a), PD-L1 (Fig. 1c), and CD8 (Fig. 1g) expression levels, but lower miR-15a level (Fig. 1b) in PC tissues than in normal tissues. Further analysis revealed a negative correlation between KCNQ1OT1 and miR-15a (Fig. 1d) and between miR-15a and PD-L1 (Fig. 1e), while a positive correlation between KCNQ1OT1 and PD-L1 (Fig. 1f), and also between CD8 and PD-L1 (Fig. 1 h) in PC tissues. The up-regulated CD8 mRNA level in PC tissues also was translated to the protein level by IHC assay, as represented by the abundance of CD8^+^ T cells in PC tissues, relative to the normal tissues (Fig. 1i). These findings support our hypothesis that KCNQ1OT1 might sponge miR-15a, releasing the control of the miR-15a on PD-L1 and thus promoting the recruitment of CD8^+^ T cells in PC tissues.

### MiR-15a negatively regulated the expression of PD-L1

**Fig. 2:**
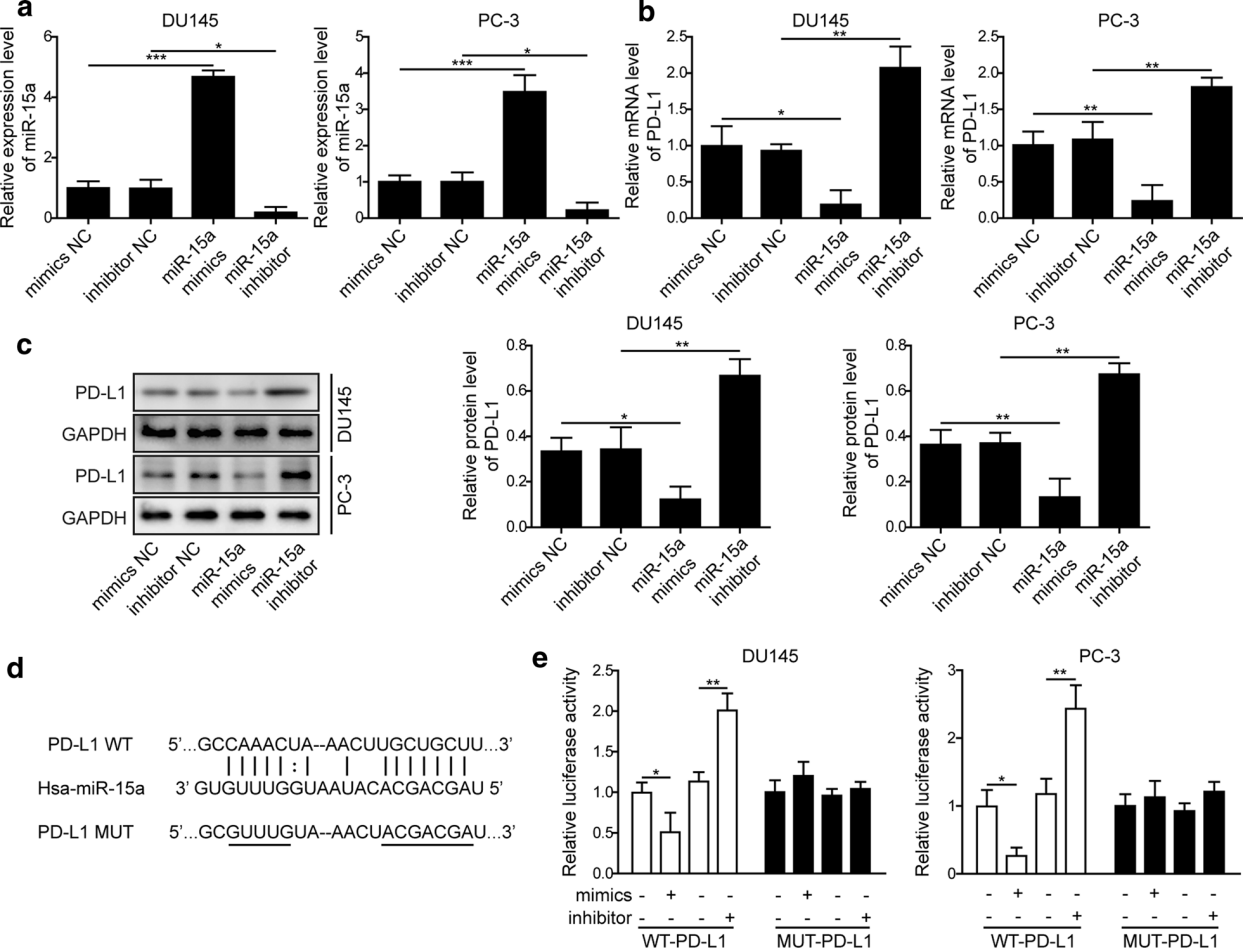
MiR-15a negatively regulated the expression of PD-L1. **a**, **b** The expression levels of miR-15a (**a**) and PD-L1 (**b**) were examined by RT-qPCR. **c** The protein level of PD-L1 was examined by western blot. DU145 and PC-3 were transfected with miR-15a mimics, miR-15a inhibitor, or the corresponding control miRNA (mimics NC or inhibitor NC). **d** Bioinformatic analysis using Starbase (http://starbase.sysu.edu.cn/index.php) revealed potential binding sites between miR-15a and PD-L1. **e** The relative luciferase activities were measured and compared between cells co-transfected with miR-15a mimics or miR-15a inhibitor. DU145 or PC-3 cells were transfected with luciferase reporter gene driven by either the wild type of PD-L1 (WT-PD-L1) or the mutated PD-L1 (MUT-PD-L1). *P < 0.05, **P < 0.01 and ***P < 0.001

An earlier study showed that miR-15a directly targeted PD-L1 in malignant pleural mesothelioma [31]. To examine whether this mechanism also remains in PC, we transfected two distinct PC cell lines DU145 and PC-3 with either miR-15a mimics or inhibitor, which significantly boosted or reduced miR-15a level, when compared to cells transfected with mimics NC or inhibitor NC (Fig. 2a). Corresponding to the altered expression of miR-15a, we detected the decreased expression of PD-L1 in cells transfected with miR-15a mimics, while the increased PD-L1 expression in those transfected with miR-15a inhibitor, both on the mRNA (Fig. 2b) and the protein (Fig. 2c) levels. Bioinformatic analysis using Starbase website revealed potential binding sites between miR-15a and PD-L1 (Fig. 2d). The direct regulation between these two molecules was further tested using the luciferase reporter assay, as shown in Fig. 2e, miR-15a mimics potently reduced, while miR-15a inhibitor boosted the luciferase reporter activity specifically driven by wild type, but not by mutant PD-L1 sequence. These results suggest that miR-15a directly inhibits PD-L1 expression in PC cells.

### PD-L1 played a central role in antagonizing miR-15a-induced cytotoxicity of CD8^+^ T cells

**Fig. 3:**
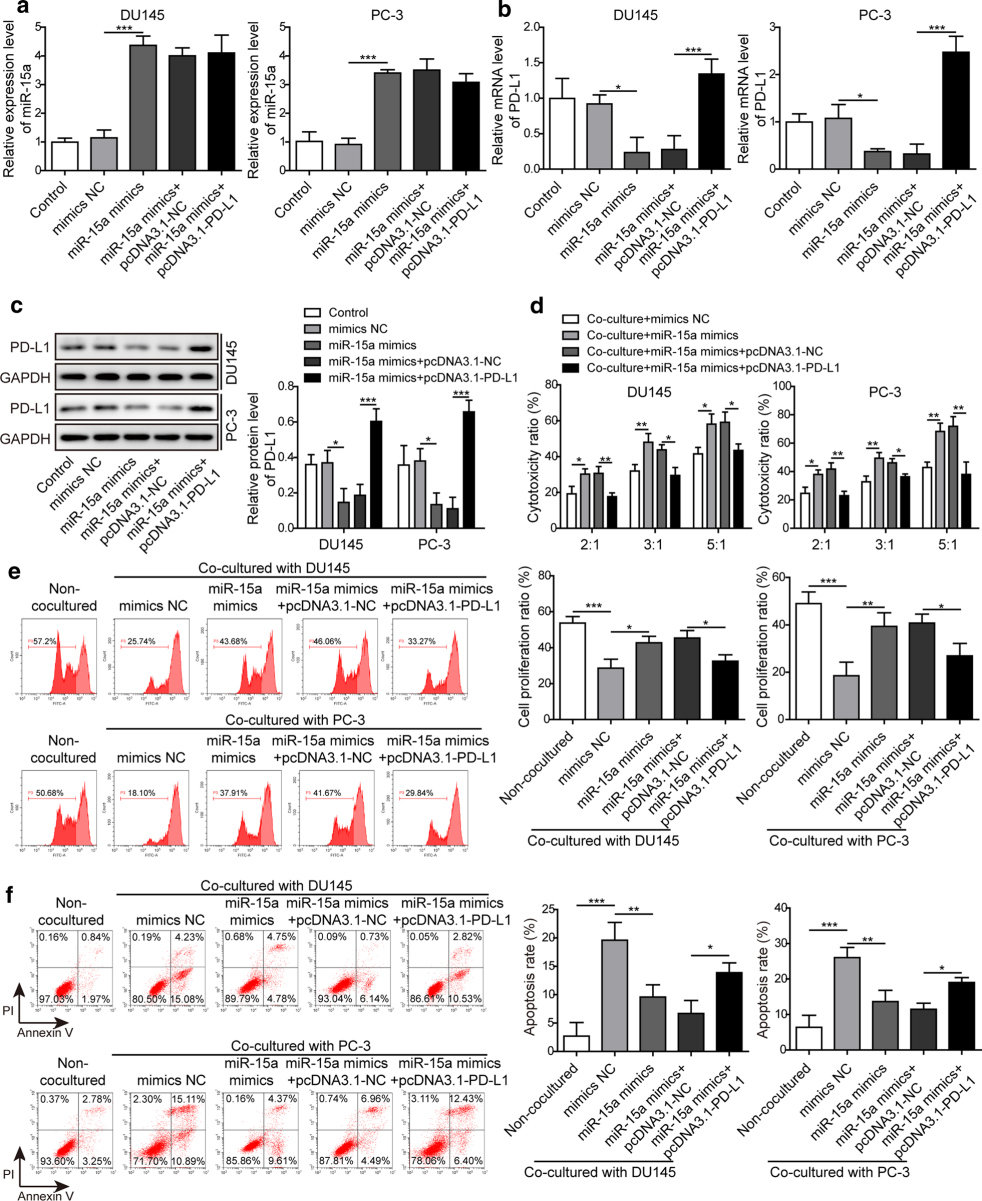
PD-L1 played a central role in antagonizing miR-15a-induced cytotoxicity of CD8^+^ T cells. **a**, **b** The expression levels of miR-15a (**a**) and PD-L1 (**b**) were examined by RT-qPCR. **c** The protein level of PD-L1 was examined by western blot. **d** The cytotoxicity of CD8^+^ T cells was measured using LDH Kit. **e** The proliferation of CD8^+^ T cells was measured by CFSE assay followed by flow cytometry. **f** The apoptosis of CD8^+^ T cells was examined by Annexin V/PI dual staining followed by flow cytometry. The effector CD8^+^ T cells were isolated from peripheral blood of healthy donors and co-cultured with indicated target PC cells. DU145 and PC-3 cells were not transfected or transfected with miR-15a mimics, miR-15a mimics + pcDNA3.1-NC, or miR-15a mimics + pcDNA3.1-PD-L1, respectively. *P < 0.05, **P < 0.01 and ***P < 0.001

Although the higher level of PD-L1 and infiltrated CD8^+^ T cells were associated with higher risk of tumor progression for node-negative PC patients, PD-L1 could also impair the functions of CD8^+^ T cells in tumors [18]. To examine whether the decreased PD-L1 expression induced by miR-15a in PC cells may impact the cytotoxicity of CD8^+^ T cells, we co-cultured CD8^+^ T cells (effector cells) with DU145 or PC-3 cells (target cells) which overexpressed miR-15a and PD-L1. By monitoring the expression levels of miR-15a and PD-L1, we found that overexpressing PD-L1 in PC cells did not impact miR-15a level (Fig. 3a), but it did significantly increase PD-L1 expression both on mRNA and protein levels (Fig. 3b, c). MiR-15a mimics significantly boosted the cytotoxicity of the CD8^+^ T cells against PC cells, yet overexpressing PD-L1 obviously abolished this effect (Fig. 3d). Consistently, we detected corresponding changes on proliferation and apoptosis of CD8^+^ T cells. The proliferation ratio was inhibited while the apoptosis ratio was increased after co-cultured with negative control PC cells (Fig. 3e, f). Transfecting DU145 or PC-3 cells with miR-15a mimics was sufficient to promote the proliferation and inhibit the apoptosis of CD8^+^ T cells, while overexpressing PD-L1 in miR-15a mimics-transfected PC cells reversed these effects (Fig. 3e, f). Taken together, these data suggest that miR-15a is an upstream regulator of PD-L1 and not vice versa. Functionally, miR-15a overexpression promotes the cytotoxicity of CD8^+^ T cells against PC cells via directly targeting PD-L1.

### MiR-15a regulated multiple malignant phenotypes of PC cells via directly targeting PD-L1

**Fig. 4:**
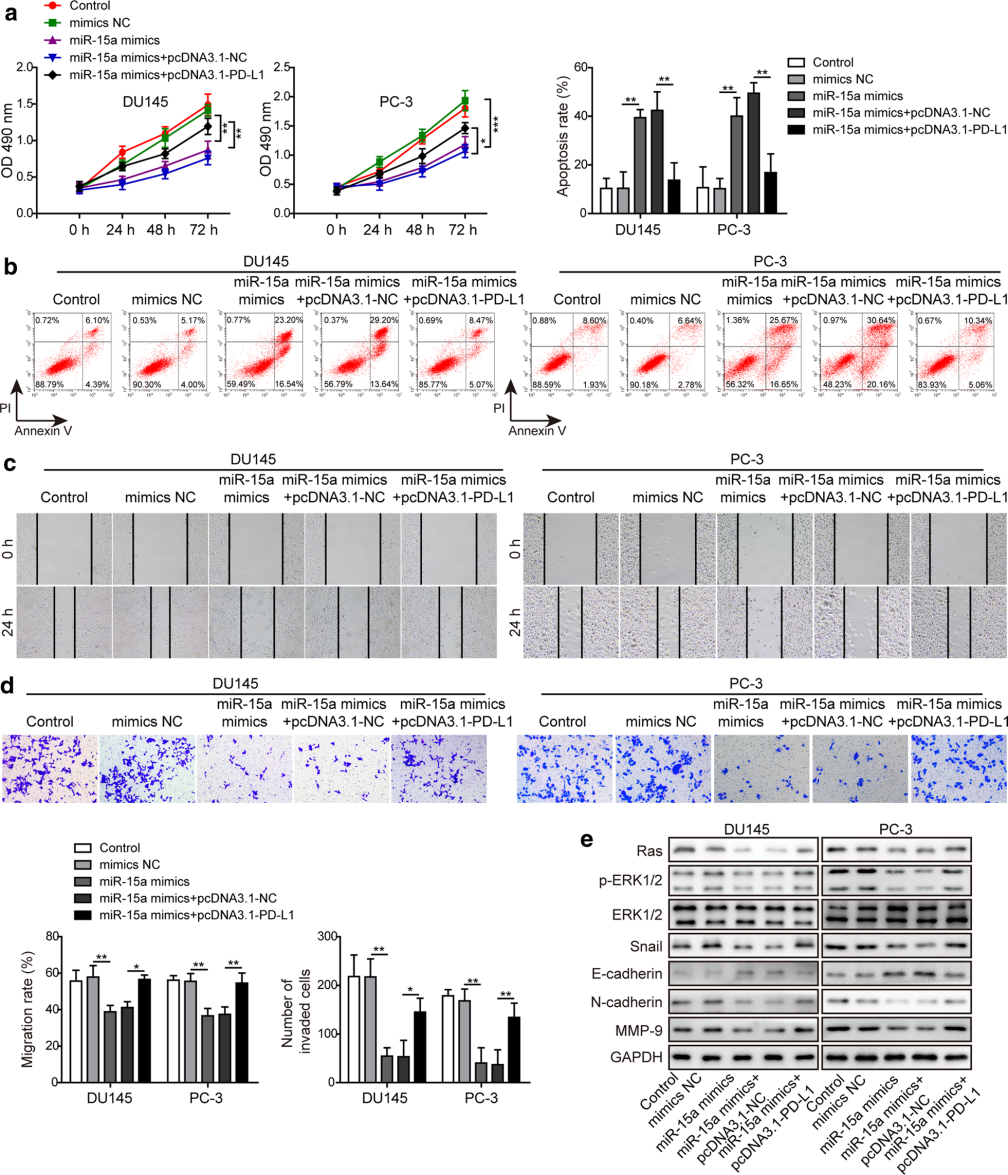
MiR-15a regulated multiple malignant phenotypes of PC cells via directly targeting PD-L1. **a**–**d** Cell viability (**a**), apoptosis (**b**), migration (**c**), and invasion (**d**) of indicated PC cells were examined by MTT assay, Annexin V/PI staining followed by flow cytometry, wound healing assay, or Transwell assay, respectively. **e** The protein levels of Ras, p-ERK1/2, ERK1/2, Snail, E-cadherin, N-cadherin, MMP-9 from indicated PC cells were examined by western blot. DU145 and PC-3 cells were not transfected or transfected with miR-15a mimics, miR-15a mimics + pcDNA3.1-NC, or miR-15a mimics + pcDNA3.1-PD-L1, respectively. *P < 0.05, **P < 0.01 and ***P < 0.001

In addition to examining the paracrine effects of the miR-15a/PD-L1 axis on cytotoxicity of CD8^+^ T cells, we also explored the autocrine effects on the cell viability, apoptosis, migration and invasion abilities of PC cells. The data showed that miR-15a overexpression was sufficient to reduce the cell viability (Fig. 4a), migration (Fig. 4c), or invasion (Fig. 4d) abilities, but increase the apoptosis (Fig. 4b) of PC cells, demonstrating the anti-cancer activities of miR-15a. In contrast, overexpressing PD-L1 in miR-15a mimics-transfected PC cells reversed the above phenotypes (Fig. 4a–d), suggesting that PD-L1 constitutes an essential mechanism for controlling the tumor-suppressive activities of miR-15a. Besides the functional phenotypes, we also examined the impacts of the miR-15a/PD-L1 axis on Ras/ERK signaling (as suggested by an earlier study [32]) and biomarker molecules involved in EMT. As shown in Fig. 4e, miR-15a mimics up-regulated E-cadherin, but down-regulated Ras, p-ERK1/2, Snail, N-cadherin, and MMP-9 levels, implying its effects on inhibiting EMT and Ras/ERK signaling. PD-L1, however, was capable of reversing the effect of miR-15a mimics on EMT and Ras/ERK signaling. Collectively, these data suggest that PD-L1 stimulates multiple malignant phenotypes by activating Ras/ERK signaling.

### KCNQ1OT1 negatively regulated the expression of miR-15a and inhibited the cytotoxicity of CD8^+^ T cells

**Fig. 5:**
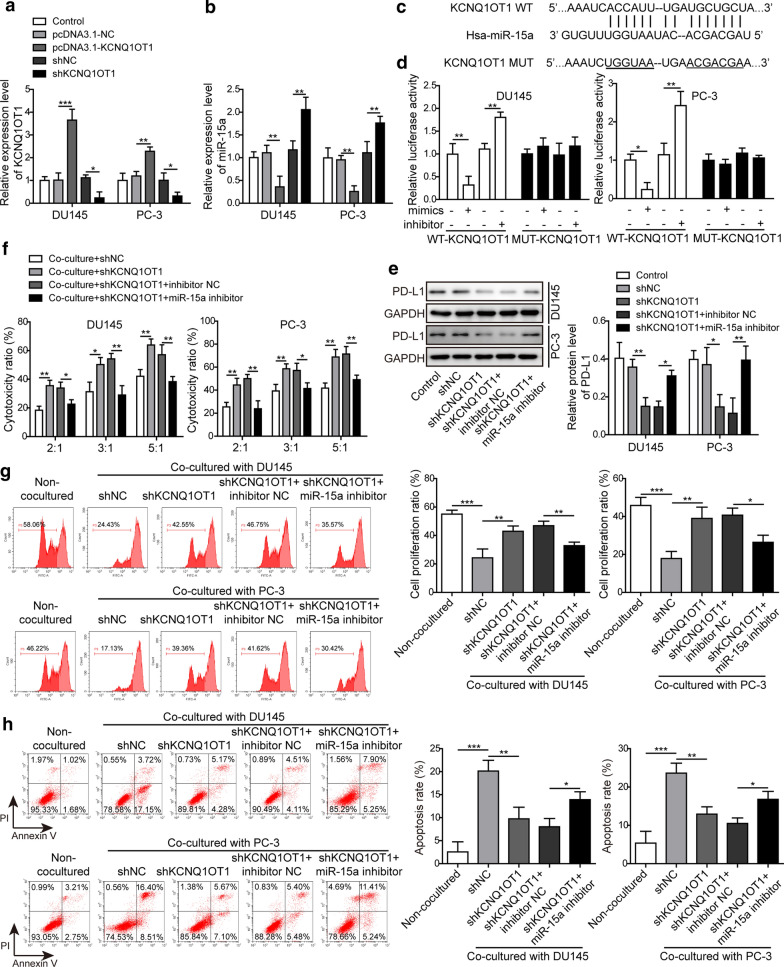
KCNQ1OT1 negatively regulated the expression of miR-15a and inhibited the cytotoxicity of CD8^+^ T cells. **a**, **b** The expression levels of KCNQ1OT1 (**a**) and miR-15a (**b**) were examined by RT-qPCR. DU145 or PC-3 cells were transfected pcDNA3.1-NC, pcDNA3.1-KCNQ1OT1, shNC, or shKCNQ1OT1. Non-transfected cells were used as control. **c** Bioinformatic analysis using Starbase (http://starbase.sysu.edu.cn/index.php) revealed potential binding sites between KCNQ1OT1 and miR-15a. **d** The relative luciferase activities were measured and compared between cells co-transfected with miR-15a mimics or miR-15a inhibitor. DU145 or PC-3 cells were transfected with luciferase reporter gene driven by either the wild type of KCNQ1OT1 (WT-KCNQ1OT1) or the mutated KCNQ1OT1 (MUT-KCNQ1OT1). **e** The protein level of PD-L1 was measured in indicated cells using western blot. **f**–**h** The cytotoxicity (**f**), proliferation (**g**), and apoptosis (**h**) of CD8^+^ T cells upon co-cultured with indicated PC cells were measured as described in Fig. 3. DU145 and PC-3 cells were not transfected or transfected with shNC, shKCNQ1OT1, shKCNQ1OT1 + inhibitor NC, or shKCNQ1OT1 + miR-15a inhibitor, respectively. *P < 0.05, **P < 0.01 and ***P < 0.001

We identified KCNQ1OT1 as the potential binding partner for miR-15a. Considering that KCNQ1OT1 promoted the progression and metastasis of various cancers [24, 26], and was up-regulated in PC tissues, and negatively correlated with miR-15a level (Fig. 1), we examined its regulation on miR-15a and the functional significance of KCNQ1OT1/miR-15a axis. We firstly adopted both gain-of-function and loss-of-function strategies, and transfected DU145 and PC-3 cells with either pcDNA3.1-KCNQ1OT1 or shKCNQ1OT1 vector. As expected, pcDNA3.1-KCNQ1OT1 transfection increased while shKCNQ1OT1 transfection reduced KCNQ1OT1 level in PC cells (Fig. 5a). Correspondingly, we detected the down-regulation of miR-15a level after KCNQ1OT1 overexpression and the up-regulation of it after KCNQ1OT1 silencing (Fig. 5b), supporting the negative regulation of miR-15a by KCNQ1OT1. Next, we introduced mutation into the potential binding sequences of KCNQ1OT1 (Fig. 5c) and then showed that miR-15a mimics specifically suppressed while miR-15a inhibitor increased the luciferase activity driven by wildtype, but not mutant KCNQ1OT1 sequences (Fig. 5d), demonstrating the direction interaction and the negative regulation between these two RNA molecules. Furthermore, we showed that knocking down KCNQ1OT1 in PC cells reduced the endogenous PD-L1 level, while simultaneously reducing miR-15a returned PD-L1 level, suggesting that KCNQ1OT1 sponged miR-15a and released the suppression of the latter on PD-L1 (Fig. 5e). Functionally, knocking down KCNQ1OT1 in PC cells enhanced the cytotoxicity of CD8^+^ T cells, while lowering miR-15a level in KCNQ1OT1-knockdown PC cells reduced the cytotoxicity of CD8^+^ T cells (Fig. 5f). Also, knocking down KCNQ1OT1 in PC cells increased the proliferation while inhibited the apoptosis of CD8^+^ T cells, which were both reversed by miR-15a inhibitor (Fig. 5g, h). These results imply that by targeting miR-15a, KCNQ1OT1 in PC cells presents paracrine effects on CD8^+^ T cells, essentially suppressing the proliferation and the cytotoxicity of CD8^+^ T cells.

### KCNQ1OT1 essentially maintained the malignant phenotypes of PC cells by targeting miR-15a

**Fig. 6:**
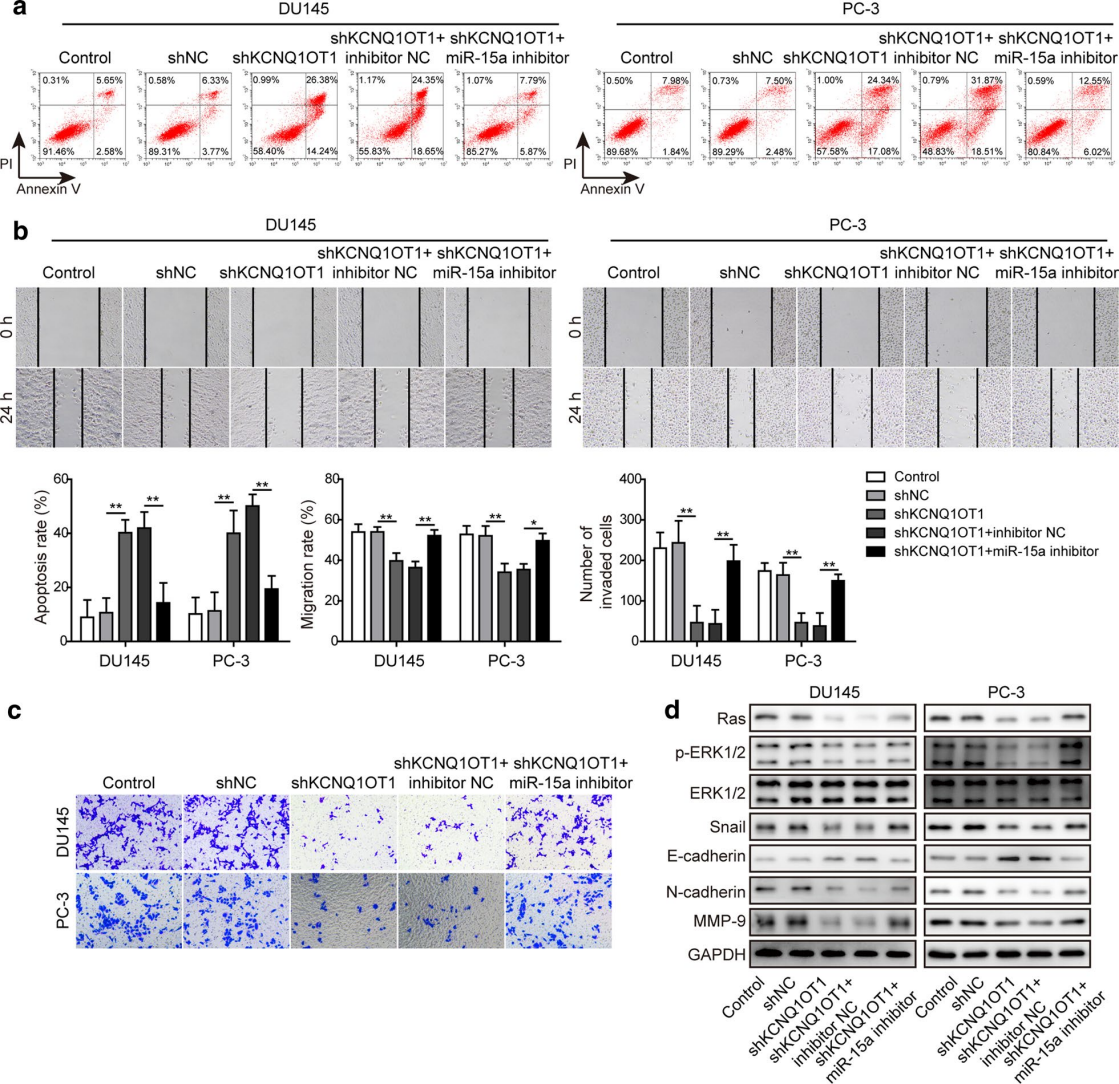
KCNQ1OT1 essentially maintained the malignant phenotypes of PC cells by targeting miR-15a. **a**–**c** The apoptosis (**a**), migration (**b**), and invasion (**c**) of indicated PC cells were examined by Annexin V/PI dual staining followed by flow cytometry, wound healing assay, and Transwell assay, respectively. **d** The protein levels of Ras, p-ERK1/2, ERK1/2, Snail, E-cadherin, N-cadherin and MMP-9 were examined by western blot. DU145 and PC-3 cells were not transfected or transfected with shNC, shKCNQ1OT1, shKCNQ1OT1 + inhibitor NC, or shKCNQ1OT1 + miR-15a inhibitor, respectively. *P < 0.05 and **P < 0.01

Lastly, we assessed the importance of KCNQ1OT1/miR-15a axis in regulating multiple malignant behaviors of PC cells. As shown in Fig. 6, knocking down KCNQ1OT1 promoted apoptosis (Fig. 6a), while inhibited the migration (Fig. 6b) and invasion abilities (Fig. 6c). Also, knocking down KCNQ1OT1 induced E-cadherin, but down-regulated Ras, p-ERK1/2, Snail, N-cadherin and MMP-9 levels in PC cells (Fig. 6d). Concomitant reducing both KCNQ1OT1 and miR-15a in PC cells abolished the effects of KCNQ1OT1 silencing alone, suggesting that KCNQ1OT1 was essential for maintaining multiple malignant phenotypes of PC cells, and targeting miR-15a allowed for the pro-tumor activities of KCNQ1OT1.

## Discussion

**Fig. 7:**
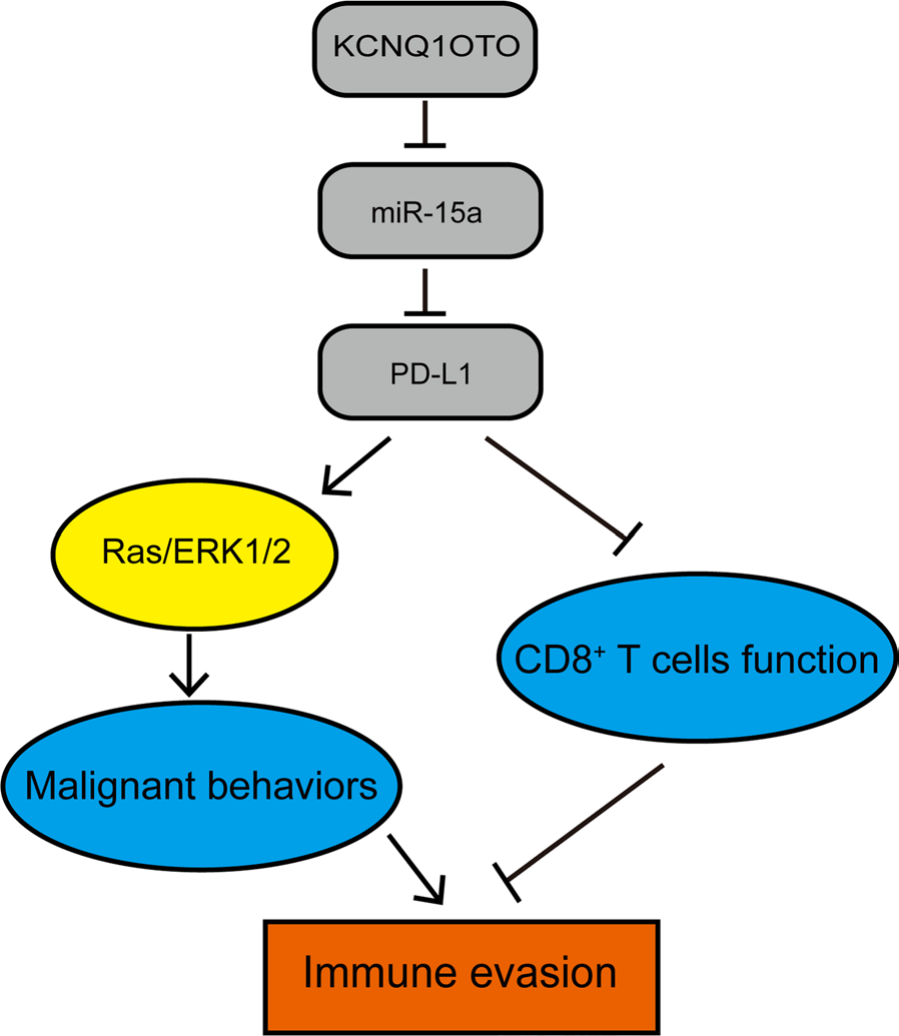
A schematic summary of this study. In PC, lncRNA KCNQ1OT1 sponges and negatively regulates miR-15a expression to up-regulate PD-L1. PD-L1, on one hand, activates Ras/ERK signaling and promotes multiple malignant phenotypes of PC cells, and on the other hand inhibits the function of CD8^+^ T cells, promoting immune evasion

Cancer development is a multistep process involving the acquirement of eight essential hallmarks “sustaining proliferative signaling, evading growth suppression, resisting cell death, enabling replicative immortality, inducing angiogenesis, activating invasion and metastasis, reprogramming of energy metabolism, and evading immune destruction” [33]. Cancer cells exploit multiple mechanisms to achieve immune evasion, including activating and generating regulatory immune cells to establish an immunosuppressive microenvironment that nurtures tumor growth, suppressing antigen-presenting machinery, producing immunosuppressive mediators, and inducing anergy or apoptosis of cytotoxic immune cells [34]. Consequently, strategies are actively sought to target these mechanisms. PD-L1/PD-1 axis plays a pivotal role in inducing anergy of cytotoxic T cells and maintaining immunosuppressive microenvironment [35], which is supported by the efficacy of antibodies targeting PD-1 or PD-L1 in non-small-cell lung cancer (NSCLC), gastric cancer, melanoma and hepatocellular carcinoma [12–14, 36, 37]. In contrast, the lack of equivalent effects in PC [15, 38] indicates that our understanding on PD-L1/PD-1 axis in PC is still quite limited. In this study, we showed for the first time that lncRNA KCNQ1OT1 sponged miR-15a to up-regulate PD-L1 expression, resulted in inhibiting the cytotoxicity of CD8^+^ T cells and promoting tumor evasion (Fig. 7).

Cumulative evidence has revealed the up-regulation of PD-L1 in PC. Gevensleben et al. reported that PD-L1 was highly expressed in aggressive primary PC and was an independent prognostic biomarker for biochemical recurrence [39, 40]. Bishop et al. found that PD-L1 was highly expressed in Enzalutamide-resistant PC [41]. Using IHC, Sharma et al. showed that positive PD-1 and PD-L1 expressions were more prevalent in PC than in normal tissues [42]. By applying IHC on tissue microarray of 535 PC specimens, Ness et al. reported that PD-L1 expression could be detected in tumor epithelial cells from 92% of the cases and the high density of PD-1^+^ lymphocytes was a negative independent prognostic factor for clinical failure-free survival [43]. Consistent with these studies, here we showed that PD-L1 was significantly up-regulated and its level positively correlated with CD8 level in PC tissues. Our subsequent experiments performed in DU145 and PC-3 cells suggested that PD-L1, when overexpressed in PC cells, was sufficient to inhibit the cytotoxicity of CD8^+^ T cells and promote the malignant behaviors of PC cells.

The expression of PD-L1 in tumor cells is regulated on multiple levels, from genetic aberrations, epigenetic regulations that involve DNA methylation, histone modification, and miRNAs, to post-translational modifications, and through both extrinsic and intrinsic oncogenic signalings [44]. Kao et al. reported that miR-15a and miR-16 down-regulated PD-L1 mRNA and protein levels in malignant pleural mesothelioma [31]. Here we also showed that miR-15a directly targeted the expression of PD-L1. More importantly, there was significantly negative association between PD-L1 and miR-15a expressions in PC tissues, supporting the clinical relevance of their crosstalk in PC development. Luciferase assay demonstrated the direct binding of miR-15a on 3′-UTR of PD-L1. Functionally, miR-15a mimics was sufficient to present anti-cancer activities by simultaneously targeting the viability, migration, invasion and EMT, promoting the apoptosis of PC cells, and enhancing the cytotoxicity and proliferation, while reducing the apoptosis of CD8^+^ T cells. All the tumor suppressive phenotypes induced by miR-15a mimics were reversed by over-expressing PD-L1, demonstrating the importance of PD-L1 in antagonizing the activities of miR-15a. To our knowledge, this was the first study showing that miR-15a could directly target PD-L1 and regulate immune evasion. In addition to PD-L1, other mechanisms were reported to mediate the tumor suppressive activities of miR-15a. Jin et al. showed that miR-15a/miR-16 cluster inhibited EMT and invasion of PC cells by targeting TGF-β signaling [45]. Bonci demonstrated that miR-15a/miR-16 cluster targeted multiple oncogenes including BCL2, CCND1 and WNT3A to induce growth arrest, apoptosis and regression of prostate xenografts [21]. Collectively, these data suggest miR-15a is a master inhibitor of PC progression and it is interesting for future study to look into the crosstalk between PD-L1 and other known signaling cascades downstream of miR-15a. The homozygous deletion of miR-15a/miR-16 gene cluster was reported in a small subset of PC samples [46], which might account for the reduced expression of miR-15a in some PC tissues.

Yet we identified that the lncRNA KCNQ1OT1 suppressed miR-15a expression in PC cells, not only their expressions negatively associated with each other in human PC tissues, but also KCNQ1OT1 directly bound to miR-15a and was sufficient and necessary to inhibit endogenous miR-15a level in PC cells. Functionally, knocking down KCNQ1OT1 lowered PD-L1 expression, inhibited the viability, migration, invasion and EMT, promoted the apoptosis of tumor cells, and enhanced the cytotoxicity and proliferation, while reduced the apoptosis of CD8^+^ T cells. These replicated the same phenotypes as miR-15a mimics and were abolished when endogenous miR-15a level was also reduced. Earlier studies showed that KCNQ1OT1 promoted NSCLC progression by modulating miRNA-27b-3p/HSP90AA1 axis [23], and stimulated cholangiocarcinoma development via miR-140-5p/SOX4 axis [47], and facilitated the migration and EMT of colorectal cancer by forming a feedback loop with miR-217 and ZEB1 [48]. Therefore, our study added, for the first time, miR-15a as a direct target for KCNQ1OT1 and the KCNQ1OT1/miR-15a axis to the oncogenic mechanisms of PC.

Several studies also have suggested upstream signaling pathways regulating KCNQ1OT1 in different disease paradigms. Shen et al. reported that YY1 up-regulated KCNQ1OT1 and contributed to atrial fibrillation [49]. Sunamura et al. showed that β-catenin promoted the development of colorectal cancer by directly up-regulating the transcription of KCNQ1OT1 [50]. Differential methylation of KCNQ1OT1 promoter polymorphism controlled its expression and associated with cardiac long QT [51]. Here we don’t know the mechanism responsible for KCNQ1OT1 up-regulation, which should be explored further in future studies. Also, since miR-16 shares the same seed sequence with miR-15a and both are derived from a gene cluster on chromosome 13q14, they may present similar functions by regulating the same set of target genes. Therefore, the novel mechanism identified for miR-15a in this study may also apply for miR-16. The axis of KCNQ1OT1/miR-16/PD-L1 in regulation of immune evasion and malignant behaviors of other cancers will be worthy of our attention.

ERK signaling promotes immune evasion of cancer cells through multiple mechanisms. In lung cancer, activated ERK1/2 signaling promoted the secretion of IL-10 and recruited immunosuppressive Tregs and M2 polarized tumor-associated macrophages to the tumor microenvironment [52]. Mutated Kras oncogene with activated ERK signaling also inhibited the NK cell surveillance program that kills tumor cells [53]. Besides, ERK signaling directly activated the transcription of PD-L1 and stabilized PD-L1 mRNA [54, 55]. In return, Qiu et al. showed that by binding to Ras and activating Ras/ERK signaling, PD-L1 stimulated multiple malignant phenotypes, including proliferation, migration and EMT of glioblastoma multiforme [32]. In this study, we demonstrated that the KCNQ1OT1/miR-15a/PD-L1 axis essentially promoted the activation of Ras/ERK signaling, which was concomitant with their stimulation of other malignant phenotypes of PC, supporting that one mechanism responsible for the pro-tumor activities of KCNQ1OT1/miR-15a/PD-L1 axis was through the activation of Ras/ERK signaling.

## Conclusions

In summary, this study reveals a novel mechanism, the KCNQ1OT1/miR-15a/PD-L1 axis simultaneously promotes multiple tumor-autonomous malignant phenotypes and inhibits the function of CD8^+^ T cells (Fig. 7). The findings suggest that up-regulating miR-15a or down-regulating KCNQ1OT1 and PD-L1 may become a promising therapy that not only targets tumor evasion, but also inhibits malignant growth of PC cells.

## Data Availability

All data generated or analyzed during this study are included in this published article.

## Abbreviations

PC: Prostate cancer
mCRPC: Metastatic castration-resistant PC
EMT: Epithelial–mesenchymal transition
PD-L1: Programmed death ligand 1
UTR: Untranslated region
LncRNA: Long non-coding RNA
RT-qPCR: Reverse transcription quantitative real-time PCR
IHC: Immunohistochemistry
DAB: Diaminobenzidine
DMEM: Dulbecco’s modified Eagle’s medium
WT: Wild type
MUT: Mutated
LDH: Lactate dehydrogenase
CFSE: Carboxyfluorescein succinimidyl ester
MTT: 3-(4, 5-dimethylthiazolyl-2)-2, 5-diphenyltetrazolium bromide
SD: Standard deviation
ANOVA: Analysis of variance
NSCLC: Non-small-cell lung cancer
